# Dynamics of sodium current mediated early afterdepolarizations

**DOI:** 10.1016/j.heliyon.2017.e00388

**Published:** 2017-09-06

**Authors:** Daisuke Sato, Colleen E. Clancy, Donald M. Bers

**Affiliations:** Department of Pharmacology, University of California, Davis, CA, United States

**Keywords:** Medicine, Applied mathematics, Systems biology, Cell biology, Physiology, Cardiology, Biophysics

## Abstract

Early afterdepolarizations (EADs) have been attributed to two primary mechanisms: 1) recovery from inactivation of the L-type calcium (Ca) channel and/or 2) spontaneous Ca release, which depolarizes the membrane potential through the electrogenic sodium-calcium exchanger (NCX). The sodium (Na) current (*I*_Na_), especially the late component of the Na current, has been recognized as an important player to set up the conditions for EADs by reducing repolarization reserve and increasing intracellular Na concentration, which leads to Ca overload. However, *I*_Na_ itself has not been considered as a direct initiator of EADs. A recent experimental study by Horvath et al. has shown that the amplitude of the late component of the Na current is as large as potassium (K) and Ca currents (∼1 pA/pF). This result suggests that *I*_Na_ by itself can exceeds the sum of outward currents and depolarize the membrane potential. In this study, we show that *I*_Na_ can also directly initiate EADs. Mathematical analysis reveals a fundamental dynamical origin of EADs arising directly from the Na channel reactivation. This system has three fixed points. The dynamics of the *I*_Na_ mediated EAD oscillation is different from that of the membrane voltage oscillation of the pacemaker cell, which has only one fixed point.

## 1 Introduction

Cardiac arrhythmia is often triggered by premature ventricular contractions (PVCs), which have been linked to early afterdepolarizations (EADs) [1, 2, 3, 4, 5]. EADs have been thought to be caused by reactivation of the L-type calcium (Ca) channel or spontaneous Ca releases from the sarcoplasmic reticulum (SR), which depolarize the membrane potential (V_m_) via the electrogenic sodium(Na)-Ca exchanger (NCX) [6, 7, 8, 9, 10, 11, 12, 13]. During the upstroke (phase 0) of the action potential (AP), Na channel opening gives large but extremely short (∼1 ms) inward current [14]. Then, immediately following the upstroke, the Na channel goes to the inactivated state. At the plateau phase of AP, the amplitude of Na current (*I*_Na_) is thought to be much smaller than the other currents and the shape and the duration of AP are mainly determined by the other currents such as the L-type Ca current (*I*_CaL_), NCX, and potassium (K) currents [15]. Na channel mutations have been associated with long QT syndrome by increasing window current and non-inactivating current [16, 17, 18]. Recent experimental measurement by Horvath et al. has shown that the amplitude of *I*_Na_ at phases 2 and 3 of AP (‘late component of the Na current’ or simply ‘late Na current’) can be surprisingly large and of similar amplitude to outward K currents [19]. This implies that the inward current via *I*_Na_ may become larger than the sum of outward currents and V_m_ can be depolarized. In this study, using a physiologically detailed model of a cardiac ventricular myocyte, we show that *I*_Na_ not only sets up the conditions for EADs by reducing repolarization reserve and increasing intracellular Na concentration, which leads to Ca overload, but also can directly initiate EADs. Mathematical reduction of the detailed model was then performed to generate 2- and 3-variable models, whose variables are membrane potential, inactivation of the Na channel, and the K conductance (for the third variable of the 3-variable model). Analysis in the reduced models reveals a fundamental dynamical origin of EADs arising directly from the Na channel reactivation, as oscillations of V_m_ at phases 2 and/or 3 of AP. Oscillatory behavior has also been extensively investigated in neuron [20, 21, 22] and pacemaker cells [23, 24]. We show that these ventricular myocyte EADs have a different dynamical mechanism from those of pacemaker cell V_m_ oscillation.

## 2 Materials and methods

### 2.1 Mathematical formulation

We use a physiologically detailed mathematical model of the rabbit ventricular action potential by Mahajan *et al.* [25]. The membrane voltage is governed by

$$\frac{dV}{dt}=-\frac{\sum I}{C_{m}}\text{,}$$

where *V* is the membrane voltage, *C_m_* is the cell capacitance, *I* represents the transmembrane currents. The details of the mathematical model are described in the next section.

There are several proposed mechanisms of the late component of *I*_Na_ [16, 17, 18]. In this study we consider two mechanisms; (1) large window current mechanism and (2) non-inactivating current mechanism.

**Fig. 1:**
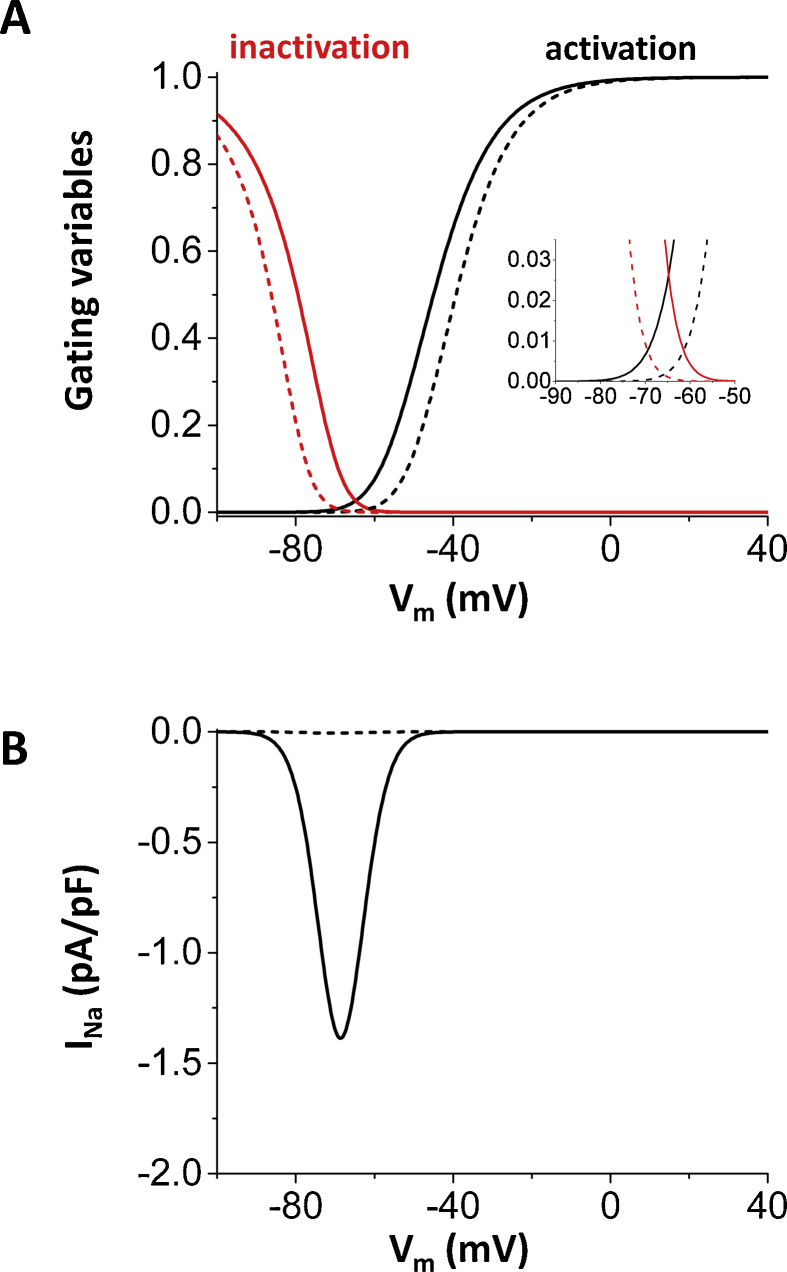
Normal Na current and pathological (increased late component of *I*_Na_) Na current. (A) Activation and inactivation curves. Activation curve is *m*^3^ and inactivation curve is *h* × *j*. Solid lines: Increased window for the late component of *I*_Na_. Dashed lines: normal Na channel. (B) Steady state current. Solid lines: Na current with the increased window. Dashed lines: normal Na current.

In order to increase the window current, activation and inactivation curves are shifted (Fig. 1A). The formula for *I*_Na_ is

$$I_{Na}=G_{Na}m^{3}hj\left(V-E_{Na}\right)\text{,}$$

where *G*_Na_ is the maximum conductance, *E*_Na_ is the reversal potential given by $RT/F\text{log}\left({\left[Na\right]}_{o}/{\left[Na\right]}_{i}\right)$, where *R* is the gas constant, *T* is the temperature, *F* is the Faraday constant, [Na]_o_ is the outside Na concentration, [Na]_i_ is the cytosolic Na concentration. *m* is the Na activation and *h* and *j* are the fast and slow Na inactivation, respectively. The window current of *I*_Na_ was increased so that the peak of the ‘window’ becomes about 2–3 percent based on the experimental observations (Fig. 1A inset) [26]. Here we note that in the major mathematical models including Mahajan et al. model, Luo-Rudy passive model (LR1) [27], Luo-Rudy dynamic model (LRd) [28], and Shannon-Bers model [29], the peak V_m_ of the window is higher (−50 to −60 mV) than that in the experimental observations (−60 to –70 mV) [26]. Therefore, we shifted the curves so that the peak V_m_ of the window correspond reasonably well with the experimental measurements [26]. In order to shift the window, activation and inactivation are changed as follows.

$$\alpha_{m}=(0.32\frac{V+127.13}{1-\text{exp}\left(-0.1(V+127.13)\right)})\text{,}$$

$$\beta_{m}=0.08\text{exp}\left(-\frac{V}{11}\right)\text{,}$$

$$\alpha_{h}=\frac{3.5\text{exp}\left(\frac{V+100}{-23}\right)}{1+\text{exp}\left(0.15\left(V+79\right)\right)}\text{,}$$

$$\beta_{h}=\left(\frac{6.0}{1+\text{exp}\left(-0.05\left(V+32\right)\right)}\right)\text{,}$$

$$\alpha_{j}=\frac{0.175\text{exp}\left(\frac{V+100}{-23}\right)}{1+\text{exp}\left(0.15\left(V+79\right)\right)}\text{,}$$

$$\beta_{j}=\left(\frac{0.3}{1+\text{exp}\left(-0.05\left(V+32\right)\right)}\right)\text{.}$$

The difference between the *h* gate and the *j* gate is the time constant. The *j* gate is 20 times slower than the *h* gate.

In order to simulate non-inactivating current, we assumed 1% of channels are non-inactivating (smallest value of h (and j) is 0.1) gates are always open regardless of V_m_). The activation curve and the inactivation curve are show in Fig. 6A.

The physiologically detailed model has 26 variables. We reduce the number of variables in order to analyze the dynamical mechanism of EADs. We show the mechanism of oscillations of V_m_ i.e. EADs using the 2-variable model and the mechanism of termination of EADs using the 3-variable model.

### 2.2 Mathematical model (detailed description)

Our base model is an AP model by Mahajan et al. [25]. The ordinary differential equations are solved by the Euler method with adaptive time step of 0.01–0.1 ms. The program codes are written in C++. Parameters are shown in Table 1, Table 2, Table 3, Table 4, Table 5, Table 6 . Equations are as follows.

**Table 1:** SR release parameters.

Parameter	Definition	Value
*τ_r_*	Spark lifetime	30 ms
*τ_a_*	NSR-JSR relaxation time	100 ms
*g_RyR_*	Release current strength	2.58 sparks cm^2^/mA
*u*	Release slope	11.3 ms^−1^
*c_sr_*	Threshold for steep release function	90 μM/l cytosol
*s*	Release function parameter	−977 μM/ms
*τ_d_*	Submembrane-myoplasm diffusion time constant	4 ms
*τ_s_*	Dyadic junction-submembrane diffusion time constant	0.5 ms

**Table 2:** Cytosolic buffering parameters.

Parameter	Definition	Value
*B_T_*	Total concentration of Troponin C	70 μmol/l cytosol
*B_SR_*	Total concentration of SR binding sites	47 μmol/l cytosol
*B_Cd_*	Total concentration of Calmodulin binding sites	24 μmol/l cytosol
*B_mem_*	Total concentration of membrane binding sites	15.0 μmol/l cytosol
*B_sar_*	Total concentration of sarcolemma binding sites	42.0 μmol/l cytosol
*k_on_^T^*	On rate for Troponin C binding	0.0327 (μM)^−1^(ms)^−1^
*k_off_^T^*	Off rate for Troponin C binding	0.0196 ms^−1^
*K_SR_*	Dissociation constant for SR binding sites	0.6 μM
*K_Cd_*	Dissociation constant for Calmodulin binding sites	7 μM
*K _mem_*	Dissociation constant for membrane binding sites	0.3 μM
*K _sar_*	Dissociation constant for sarcolemma binding sites	13.0 μM

**Table 3:** Exchanger, uptake, and SR leak parameters.

Parameter	Definition	Value
*c_up_*	Uptake threshold	0.5 μM
*v_up_*	Strength of Uptake	0.4 μM/ms
*g_NaCa_*	Strength of exchange current	0.84 μM/s
*k_sat_*	constant	0.2
*ξ*	constant	0.35
*K_m,Nai_*	constant	12.3 mM
*K_m,Nao_*	constant	87.5 mM
*K_m,Cai_*	constant	0.0036 mM
*K_m,Cao_*	constant	1.3 mM
*c_naca_*	constant	0.3 μM
*g_l_*	Strength of leak current	2.07 × 10^−5^ (ms)^−1^
*k_j_*	Threshold for leak onset	50 μM

**Table 4:** L-type Ca current parameters.

Parameter	Definition	Value
*P_Ca_*	Constant	0.00054 cm/s
*g_Ca_*	Strength of Ca current flux	182 mmol/(cm C)
${\bar{g}}_{\mathrm{Ca}}$	Strength of local Ca flux due to L-type Ca channels	9000 mmol/(cm C)
${\bar{g}}_{SR}$	Strength of local Ca flux due to RyR channels	26842 mmol/(cm C)
*k_p_°*	Threshold for Ca-induced inactivation	3.0 μM
${\bar{\text{c}}}_{\text{p}}$	Threshold for Ca dependence of transition rate k_6_	6.1 μM
*τ_po_*	Time constant of activation	1 ms
*r_1_*	Opening rate	0.3 ms^−1^
*r_2_*	Closing rate	3 ms^−1^
*s_1_′*	Inactivation rate	0.00195 ms^−1^
*k_1_′*	Inactivation rate	0.00413 ms^−1^
*k_2_*	Inactivation rate	0.0001 ms^−1^
*k_21_′*	Inactivation rate	0.00224 ms^−1^
*T_Ba_*	Time constant	450 ms

**Table 5:** Physical constants and ionic concentrations.

Parameter	Definition	Value
*C_m_*	Cell capacitance	3.1 × 10^−4^ μF
*v_i_*	Cell volume	2.58 × 10^−5^ μl
*v_s_*	Submembrane volume	0.02 *v_i_*
*F*	Faraday constant	96.5C/mmol
*R*	Universal gas constant	8.315 J mol^−1^K^−1^
*T*	Temperature	308 K
[Na^+^]_o_	External sodium concentration	136 mM
[K^+^]_i_	Internal potassium concentration	140 mM
[K^+^]_o_	External potassium concentration	5.4 mM
[Ca^2+^]_o_	External calcium concentration	1800 μM

**Table 6:** Ion current conductances.

Parameter	Definition	Value
*g_Na_*	Peak *I_Na_* conductance	12.0 mS/μF
*g_to,f_*	Peak *I_to,f_* conductance	0.11 mS/μF
*g_to,s_*	Peak *I_to,s_* conductance	0.04 mS/μF
*g_K1_*	Peak *I_K1_* conductance	0.3 mS/μF
*g_Kr_*	Peak *I_Kr_* conductance	0.0125 mS/μF
*g_Ks_*	Peak *I_Ks_* conductance	0.1386 mS/μF
*g_NaK_*	Peak *I_NaK_* conductance	1.5 mS/μF

### 2.3 Ionic currents

The membrane voltage (*V_m_*) is given by

$$\frac{dV}{dt}=-\frac{I_{ion}+I_{stim}}{C_{m}}\text{,}$$

where C_m_ = 1 μF/cm^2^ is membrane capacitance, *I*_ion_ is total ionic current density across the cell membrane, and *I*_stim_ is the stimulus current. The total membrane current is given by

*I*_ion_ = *I*_Na_ + *I*_to,f_ + *I*_to,s_ + *I*_Kr_ + *I*_Ks_ + *I*_K1_ + *I*_NaK_ + *I*_Ca_ + *I*_NaCa_

### 2.4 The sodium current (*I*_Na_)

We modified the original Mahajan formulation so that the peak V_m_ of the window corresponds reasonably well with the experimental measurements [26].

*I*_Na_ is given by

$$I_{Na}=G_{Na}m^{3}hj\left(V-E_{Na}\right)\text{,}$$

Na channel activation for the large window current is given by

$$\frac{dm}{dt}=(m_{\infty }-m)/\tau_{m}\text{,}$$

$$m_{\infty }=\frac{\alpha_{m}}{\alpha_{m}+\beta_{m}}\text{,}$$

$$\tau_{m}=\frac{1}{\alpha_{m}+\beta_{m}}\text{,}$$

$$\alpha_{m}=(0.32\frac{V+102.13}{1-\text{exp(}-0.1(V+102.13)\text{)}})\text{,}$$

$$\beta_{m}=0.08\text{exp}\left(-\frac{V}{11}\right)\text{,}$$

Na channel inactivation for the large window current is given by

$$\frac{dh}{dt}=(h_{\infty }-h)/\tau_{h}\text{,}$$

$$h_{\infty }=\frac{\alpha_{h}}{\alpha_{h}+\beta_{h}}\text{,}$$

$$\tau_{h}=\frac{1}{\alpha_{h}+\beta_{h}}\text{,}$$

$$\alpha_{h}=\frac{3.5\text{exp}\left(\frac{V+95}{-23}\right)}{1+\text{exp}\left(0.15\left(V+74\right)\right)}\text{,}$$

$$\beta_{h}=\left(\frac{6.0}{1+\text{exp}\left(-0.05\left(V+32\right)\right)}\right)\text{,}$$

$$\frac{dj}{dt}=(j_{\infty }-j)/\tau_{j}\text{,}$$

$$j_{\infty }=\frac{\alpha_{j}}{\alpha_{j}+\beta_{j}}\text{,}$$

$$\tau_{j}=\frac{1}{\alpha_{j}+\beta_{j}}\text{,}$$

$$\alpha_{j}=\frac{0.175\text{exp}\left(\frac{V+95}{-23}\right)}{1+\text{exp}\left(0.15\left(V+74\right)\right)}\text{,}$$

$$\beta_{j}=\left(\frac{0.3}{1+\text{exp}\left(-0.05\left(V+32\right)\right)}\right)\text{.}$$

On the other hand, normal Na channel activation is given by

$$\frac{dm}{dt}=(m_{\infty }-m)/\tau_{m}\text{,}$$

$$m_{\infty }=\frac{\alpha_{m}}{\alpha_{m}+\beta_{m}}\text{,}$$

$$\tau_{m}=\frac{1}{\alpha_{m}+\beta_{m}}\text{,}$$

$$\alpha_{m}=\left(0.32\frac{v+72.13}{1-\text{exp}\left(-0.1\left(V+72.13\right)\right)}\right)\text{,}$$

$$\beta_{m}=0.08\text{exp}\left(-\frac{V}{11}\right)\text{,}$$

Normal Na channel inactivation is given by

$$\frac{dh}{dt}=(h_{\infty }-h)/\tau_{h}\text{,}$$

$$h_{\infty }=\frac{\alpha_{h}}{\alpha_{h}+\beta_{h}}\text{,}$$

$$\tau_{h}=\frac{1}{\alpha_{h}+\beta_{h}}\text{,}$$

$$\alpha_{h}=\frac{3.5\text{exp}\left(\frac{V+105}{-23}\right)}{1+\text{exp}\left(0.15\left(V+84\right)\right)}\text{,}$$

$$\beta_{h}=\left(\frac{6.0}{1+\text{exp}\left(-0.05\left(V+32\right)\right)}\right)\text{,}$$

$$\frac{dj}{dt}=(j_{\infty }-j)/\tau_{j}\text{,}$$

$$j_{\infty }=\frac{\alpha_{j}}{\alpha_{j}+\beta_{j}}\text{,}$$

$$\tau_{j}=\frac{1}{\alpha_{j}+\beta_{j}}\text{,}$$

$$\alpha_{j}=\frac{0.175\text{exp}\left(\frac{V+105}{-23}\right)}{1+\text{exp}\left(0.15\left(V+84\right)\right)}\text{,}$$

$$\beta_{j}=\left(\frac{0.3}{1+\text{exp}\left(-0.05\left(V+32\right)\right)}\right)\text{.}$$

### 2.5 Inward rectifier K^+^ current (*I*_K1_)

*I*_K1_ is given by

$$I_{K1}=g_{K1}\sqrt{\frac{{\left[K^{+}\right]}_{o}}{5.4}}\frac{A_{K1}}{A_{K1}+B_{K1}}\left(V-E_{K}\right)$$

$$A_{K1}=\frac{1.02}{1.0+e^{0.2385\left(V-E_{K}-59.215\right)}}$$

$$B_{K1}=\frac{0.49124e^{0.08032\left(V-E_{K}+5.476\right)}+e^{0.061750\left(V-E_{K}-594.31\right)}}{1+e^{-0.5143\left(V-E_{K}+4.753\right)}}$$

$$E_{K}=\frac{RT}{F}\ln\left(\frac{{\left[K^{+}\right]}_{o}}{{\left[K^{+}\right]}_{i}}\right)\text{.}$$

### 2.6 The rapid component of the delayed rectifier K^+^ current (*I*_Kr_)

*I*_Kr_ is given by

$$I_{Kr}=g_{Kr}\sqrt{\frac{{\left[K^{+}\right]}_{o}}{5.4}}x_{Kr}R\left(V\right)\left(V-E_{K}\right)$$

$$R\left(V\right)=\frac{1}{1+e^{\left(V+33\right)/22.4}}$$

$$\frac{dx_{Kr}}{dt}=\frac{x_{Kr}^{\infty }-x_{Kr}}{\tau_{Kr}}$$

$$x_{Kr}^{\infty }=\frac{1}{1+e^{-\left(V+50\right)/7.5}}$$

$$\tau_{Kr}=\frac{1}{\left(\frac{0.00138\left(V+7\right)}{1-e^{-0.123\left(V+7\right)}}+\frac{0.00061\left(V+10\right)}{-1+e^{0.145\left(V+10\right)}}\right)}$$

### 2.7 The slow component of the delayed rectifier K^+^ current (*I*_Ks_)

*I*_Ks_ is given by

$$I_{Ks}=g_{Ks}x_{s1}x_{s2}q_{Ks}\left(V-E_{Ks}\right)$$

$$q_{Ks}=1+\frac{0.8}{\left(1+{\left(\frac{0.5}{c_{i}}\right)}^{3}\right)}$$

$$\frac{dx_{s1}}{dt}=\frac{x_{x}^{\infty }-x_{s1}}{\tau_{xs1}}$$

$$\frac{dx_{s2}}{dt}=\frac{x_{x}^{\infty }-x_{s2}}{\tau_{xs2}}$$

$$x_{s}^{\infty }=\frac{1}{1+e^{-\left(V-1.5\right)/16.7}}$$

$$\tau_{xs1}=\frac{1}{\left(\frac{0.0000719\left(V+30\right)}{1-e^{-0.148\left(V+30\right)}}+\frac{0.00031\left(V+30\right)}{-1+e^{0.0687\left(V+30\right)}}\right)}$$

$$\tau_{xs2}=4\tau_{xs1}$$

$$E_{Ks}=\frac{RT}{F}\ln\left(\frac{{\left[K^{+}\right]}_{o}+0.01833{\left[Na^{+}\right]}_{o}}{{\left[K^{+}\right]}_{i}+0.01833{\left[Na^{+}\right]}_{i}}\right)\text{.}$$

### 2.8 The NaK exchanger current (*I*_NaK_)

I_NaK_ is given by

$$\sigma =\frac{e^{{\left[Na^{+}\right]}_{o}/67.3}-1}{7}$$

$$f_{NaK}=\frac{1}{1+0.1245e^{-0.1VF/RT}+0.0365\sigma e^{-VF/RT}}$$

$$I_{NaK}=g_{NaK}f_{NaK}\left(\frac{1}{1+\left(12mM/{\left[Na^{+}\right]}_{i}\right)}\right)\left(\frac{{\left[K^{+}\right]}_{o}}{{\left[K^{+}\right]}_{o}+1.5mM}\right)\text{.}$$

### 2.9 The fast component of the rapid inward K^+^ current (*I*_to,f_)

*I*_to,f_ is given by

$$I_{to,f}=g_{to,f}X_{to,f}Y_{to,f}\left(V-E_{K}\right)$$

$$X_{to,f}^{\infty }=\frac{1}{1+e^{-\left(V+3\right)/15}}$$

$$Y_{to,f}^{\infty }=\frac{1}{1+e^{\left(V+33.5\right)/10}}$$

$$\tau_{Xto,f}=3.5e^{-\left(V/30\right)}+1.5$$

$$\tau_{Yto,f}=\frac{20}{1+e^{\left(V+33.5\right)/10}}+20$$

$$\frac{dX_{to,f}}{dt}=\frac{X_{to,f}^{\infty }-X_{to,f}}{\tau_{Xto,f}}$$

$$\frac{dY_{to,f}}{dt}=\frac{Y_{to,f}^{\infty }-Y_{to,f}}{\tau_{Yto,f}}$$

### 2.10 The slow component of the rapid outward K^+^ current (*I*_to,s_)

*I*_to,s_ is given by

$$I_{to,s}=g_{to,s}X_{to,s}\left(Y_{to,s}+0.5R_{s}^{\infty }\right)\left(V-E_{K}\right)$$

$$R_{s}^{\infty }=\frac{1}{1+e^{\left(V+33.5\right)/10}}$$

$$X_{to,s}^{\infty }=\frac{1}{1+e^{-\left(V+3\right)/15}}$$

$$Y_{to,s}^{\infty }=\frac{1}{1+e^{\left(V+33.5\right)/10}}$$

$$\tau_{Xto,s}=9/\left(1+e^{\left(V+3\right)/15}\right)+0.5$$

$$\tau_{Yto,s}=\frac{3000}{1+e^{\left(V+60\right)/10}}+30$$

$$\frac{dX_{to,s}}{dt}=\frac{X_{to,s}^{\infty }-X_{to,s}}{\tau_{Xto,f}}$$

$$\frac{dY_{to,s}}{dt}=\frac{Y_{to,s}^{\infty }-Y_{to,s}}{\tau_{Yto,s}}$$

### 2.11 Equations for Ca cycling

The equations for Ca cycling are:

$$\frac{dc_{s}}{dt}=\beta_{s}\left[\frac{v_{i}}{v_{s}}\left(J_{rel}-J_{d}+J_{Ca}+J_{NaCa}\right)-J_{trpn}^{s}\right]\text{,}$$

$$\frac{dc_{i}}{dt}=\beta_{i}\left[J_{d}-J_{up}+J_{leak}-J_{trpn}^{i}\right]\text{,}$$

$$\frac{dc_{j}}{dt}=-J_{rel}+J_{up}-J_{leak}\text{,}$$

$$\frac{dc_{j}^{'}}{dt}=\frac{c_{j}-c_{j}^{'}}{\tau_{a}}\text{,}$$

$$\frac{dJ_{rel}}{dt}=N_{s}^{'}\left(t\right)Cc_{j}C\frac{Q\left(c_{j}^{'}\right)}{c_{sr}}-\frac{J_{rel}}{T}\text{,}$$

$$T=\frac{\tau_{r}}{1-\tau_{r}\frac{\frac{dc_{j}}{dt}}{c_{j}}}\text{,}$$

where *c_s_*, *c_i_*, and *c_j_* are free [Ca] in the submembrane space, the cytosol, and the SR, with volumes *v_s_*, *v_i_* and *v_sr_*respectively. The concentrations *c_s_* and *c_i_*are in units of *μM*, whereas *c_j_* and *c_j_*’ (for simplicity) are both in units of μM*v_sr_/v_i_* (*μ*M/l cytosol). The current fluxes are: *J_rel_*, the total release flux out of the SR via RyR channels; *J_d_*, diffusion of Ca from the submembrane space to the bulk myoplasm; *J_up_*, the uptake current via SERCA pumps in the SR; *J_Ca_*, the current flux into the cell via L-type Ca channels; *J_NaCa_*, the current flux into the cell via the NaCa exchanger; *J_leak_*, the leak current from the SR into the bulk myoplasm. All Ca fluxes are divided by *v_i_* and have units of μM/ms, which can be converted to units of μA/μF using the conversion factor *nFv_i_/C_m_*, where *n* is the ionic charge of the current carrier, *C_m_* is the cell membrane capacitance, and where F is Faraday's constant. Ionic fluxes can be converted to membrane currents using

$$I_{Ca}=-2\alpha J_{Ca},\;I_{NaCa}=\alpha J_{NaCa}\text{,}$$

where α *= Fv_i_/C_m_*, and where the ion currents are in units of μA/μF.

The dependence of Ca release on SR Ca load is given by

$$Q\left(c_{j}^{'}\right)=\left\{ \begin{array}{c} \;\;\;\;\;0,\;\;\;\;\;\;\;0<c_{j}^{'}<50,\\ c_{j}^{'}-50,\;\;\;50\leq c_{j}^{'}\leq c_{sr},\\ uc_{j}^{'}+s,\;\;\;\;\;\;\;\;\;c_{j}^{'}>c_{sr}, \end{array}\right.$$

where the parameter *u* controls the slope of the SR Ca release vs. SR Ca load relationship at high loads (*c_j_*’ > *c_sr_*). The parameter *s* is chosen so that the function *Q(c_j_*’) is continuous at *c_sr_*.

The number of sparks recruited over the whole cell in a time interval *Δt* is given by *ΔN_s_*, and the rate of spark recruitment is *N_s_*’ *= ΔN_s_*/*Δt*. Since spark recruitment is initiated by the stochastic single channel opening of L-type Ca channels distributed throughout the cell, *N_s_*’ follows a voltage dependence similar to the whole cell Ca entry. A phenomenological expression for spark rate is given by

$$N_{s}^{'}=-g_{RyR}\left(V\right)P_{o}i_{Ca}\text{,}$$

where *g*(*V*) is the gain function, which controls the voltage dependence of Ca released into the SR in response to a trigger from the L-type Ca current. The voltage dependence is weak and has the form

$$g_{RyR}\left(V\right)=g_{RyR}\frac{e^{-0.05\left(V+30\right)}}{1+e^{-0.05\left(V+30\right)}}\text{.}$$

### 2.12 The L-type Ca current flux

The Ca flux into the cell due to the L-type Ca current is given by

$$J_{Ca}=g_{Ca}P_{o}i_{Ca}\text{,}$$

$$i_{Ca}=\frac{4P_{Ca}VF^{2}}{RT}\frac{c_{s}e^{2a}-0.341{\left[Ca^{2+}\right]}_{o}}{e^{2a}-1}\text{,}$$

where α *= VF/RT*, and where *c_s_* is the submembrane concentration in units of mM.

### 2.13 Markov model of the L-type Ca current

The equations for the Markov states of L-type Ca channels are:

$$\frac{dC_{2}}{dt}=\beta C_{1}+k_{5}I_{2Ca}+k_{5}^{'}I_{2Ba}-\left(k_{6}+k_{6}^{'}+\alpha \right)C_{2}\text{,}$$

$$\frac{dC_{1}}{dt}=\alpha C_{2}+k_{2}I_{1Ca}+k_{2}^{'}I_{1Ba}+r_{2}P_{o}-\left(r_{1}+\beta +k_{1}+k_{1}^{'}\right)C_{1}\text{,}$$

$$\frac{dI_{1Ca}}{dt}=k_{1}C_{1}+k_{4}I_{2Ca}+s_{1}P_{o}-\left(k_{2}+k_{3}+s_{2}\right)I_{1Cao}\text{,}$$

$$\frac{dI_{2Ca}}{dt}=k_{3}I_{1Ca}+k_{6}C_{2}-\left(k_{4}+k_{5}\right)I_{2Ca}\text{,}$$

$$\frac{dI_{1Ba}}{dt}=k_{1}^{'}C_{1}+k_{4}^{'}I_{2Ba}+s_{1}^{'}P_{o}-\left(k_{2}^{'}+k_{3}^{'}+s_{2}^{'}\right)I_{1Ba}\text{,}$$

$$\frac{dI_{2Ba}}{dt}=k_{3}^{'}I_{1Ba}+k_{6}^{'}C_{2}-\left(k_{5}^{'}+k_{4}^{'}\right)I_{2Ba}\text{,}$$

where the open probability satisfies

$$P_{o}=1-\left(C_{1}+C_{2}+I_{1Ca}+I_{2Ca}+I_{1Ba}+I_{2Ba}\right)\text{.}$$

The rates are given by:

$$\alpha =p_{o}^{\infty }/\tau_{po}\text{,}$$

$$\beta =\left(1-p_{o}^{\infty }\right)/\tau_{po}\text{,}$$

$$p_{o}^{\infty }=\frac{1}{1+e^{-V/8}}\text{,}$$

$$s_{1}=0.02f\left(c_{p}\right)\text{,}$$

$$k_{1}=0.03f\left(c_{p}\right)\text{,}$$

$$s_{2}=s_{1}\left(k_{2}/k_{1}\right)\left(r_{1}/r_{2}\right)\text{,}$$

$$s_{2}^{'}=s_{1}^{'}\left(k_{2}^{'}/k_{1}^{'}\right)\left(r_{1}/r_{2}\right)\text{,}$$

$$f\left(c_{p}\right)=\frac{1}{1+{\left(\frac{\tilde{c_{p}}}{c_{p}}\right)}^{3}}$$

$$k_{3}=\frac{e^{-\left(V+40\right)/3}}{3\left(1+e^{-\left(V+40\right)/3}\right)}\text{,}$$

$$k_{3}^{'}=k_{3}\text{,}$$

$$k_{4}=k_{3}\left(\alpha /\beta \right)\left(k_{1}/k_{2}\right)\left(k_{5}/k_{6}\right)\text{,}$$

$$k_{4}^{'}=k_{3}^{'}\left(\alpha /\beta \right)\left(k_{1}^{'}/k_{2}^{'}\right)\left(k_{5}^{'}/k_{6}^{'}\right)\text{,}$$

$$k_{5}=\left(1-P_{s}\right)/\tau_{Ca}\text{,}$$

$$k_{6}=f\left(c_{p}\right)P_{s}/\tau_{Ca}\text{,}$$

$$k_{5}^{'}=\left(1-P_{s}\right)/\tau_{Ba}\text{,}$$

$$k_{6}^{'}=P_{s}/\tau_{Ba}\text{,}$$

$$\tau_{Ca}=\left(R\left(V\right)-T_{Ca}\right)P_{r}+T_{Ca}$$

$$\tau_{Ba}=\left(R\left(V\right)-T_{Ba}\right)P_{r}+T_{Ba}$$

$$T_{Ca}=\frac{114}{1+{\left(\frac{c_{p}}{\bar{c_{p}}}\right)}^{4}}$$

$$R\left(V\right)=10+4954e^{V/15.6}$$

$$P_{r}=\frac{e^{-\left(V+40\right)/4}}{1+e^{-\left(V+40\right)/4}}$$

$$P_{s}=\frac{e^{-\left(V+40\right)/11.32}}{1+e^{-\left(V+40\right)/11.32}}$$

### 2.14 Diffusive flux

The flux of Ca from the submembrane space to the bulk myoplasm is given by:

$$J_{d}=\frac{c_{s}-c_{i}}{\tau_{d}}\text{,}$$

where τ_d_ is the time constant for Ca diffusion from the submembrane space to the bulk myoplasm.

### 2.15 Nonlinear buffering

Buffering of Ca is modeled by incorporating instantaneous buffering to SR, calmodulin, membrane and sarcolemma binding sites.

$$\beta_{s}={\left(1+\frac{B_{SR}K_{SR}}{{\left(c_{s}+K_{SR}\right)}^{2}}+\frac{B_{cd}K_{cd}}{{\left(c_{s}+K_{cd}\right)}^{2}}+\frac{B_{mem}K_{mem}}{{\left(c_{s}+K_{mem}\right)}^{2}}+\frac{B_{sar}K_{sar}}{{\left(c_{s}+K_{sar}\right)}^{2}}\right)}^{-1}\text{,}$$

$$\beta_{i}={\left(1+\frac{B_{SR}K_{SR}}{{\left(c_{i}+K_{SR}\right)}^{2}}+\frac{B_{cd}K_{cd}}{{\left(c_{i}+K_{cd}\right)}^{2}}+\frac{B_{mem}K_{mem}}{{\left(c_{i}+K_{mem}\right)}^{2}}+\frac{B_{sar}K_{sar}}{{\left(c_{i}+K_{sar}\right)}^{2}}\right)}^{-1}\text{.}$$

Time dependent buffering to Troponin C is described by

$$\frac{d{\left[CaT\right]}_{i}}{dt}=J_{trpn}^{i}\text{,}$$

$$\frac{d{\left[CaT\right]}_{s}}{dt}=J_{trpn}^{s}\text{,}$$

$$J_{trpn}^{i}=k_{on}^{T}c_{i}\left(B_{T}-{\left[CaT\right]}_{i}\right)-k_{off}^{T}{\left[CaT\right]}_{i}$$

$$J_{trpn}^{s}=k_{on}^{T}c_{s}\left(B_{T}-{\left[CaT\right]}_{s}\right)-k_{off}^{T}{\left[CaT\right]}_{s}\text{.}$$

### 2.16 NCX flux

The equation of the NCX is given by

$$J_{NaCa}=g_{naca}K_{a}\frac{e^{\varsigma a}{\left[Na^{+}\right]}_{i}^{3}{\left[Ca^{2+}\right]}_{o}-e^{\left(\varsigma -1\right)a}{\left[Na^{+}\right]}_{o}^{3}c_{s}}{\left(1+k_{sat}e^{\left(\varsigma -1\right)a}\right)H}$$

where

$$H=K_{m,Cao}{\left[Na^{+}\right]}_{i}^{3}+K_{m,Nao}^{3}Cc_{s}+K_{m,Nai}^{3}{\left[Ca^{2+}\right]}_{o}\left(1+\frac{c_{s}}{K_{m,Cai}}\right)+K_{m,Cai}{\left[Na^{+}\right]}_{i}^{3}\left(1+\frac{{\left[Na^{+}\right]}_{i}^{3}}{K_{m,Nai}^{3}}\right)+{\left[Na^{+}\right]}_{i}^{3}{\left[Ca^{2+}\right]}_{o}+{\left[Na^{+}\right]}_{i}^{3}c_{s}\text{,}$$

and where

$$K_{a}=\frac{1}{1+{\left(\frac{c_{naca}}{c_{s}}\right)}^{3}}\text{.}$$

### 2.17 The SERCA (uptake) pump

The SERCA Ca pump is given by

$$J_{up}=\frac{v_{up}c_{i}^{2}}{c_{i}^{2}+c_{up}^{2}}\text{,}$$

where *v_up_* denotes the strength of uptake and *c_up_*is the pump threshold.

### 2.18 The SR leak flux

The leak flux from the SR is given by

$$J_{leak}=g_{l}L\left(c_{j}\right)\left(\left(\frac{v_{i}}{v_{sr}}\right)c_{j}-c_{i}\right)\text{,}$$

where *v_sr_/v_i_* is the SR to cytoplasm volume ratio, and *L*(*c_j_*) is a threshold function of the form

$$L\left(c_{j}\right)=\frac{c_{j}^{2}}{c_{j}^{2}+k_{j}^{2}}$$

### 2.19 Ca dynamics in the dyadic space

The average concentration in active dyadic clefts is given by

$$\frac{dc_{p}}{dt}={\tilde{J}}_{SR}+{\tilde{J}}_{Ca}-\frac{c_{p}-c_{s}}{\tau_{s}}\text{,}$$

where,

$${\tilde{J}}_{Ca}=-{\bar{g}}_{Ca}P_{o}i_{Ca}\text{,}$$

$${\tilde{J}}_{SR}=-g_{SR}\left(V\right)Q\left(c_{j}^{'}\right)P_{o}i_{Ca}\text{,}$$

$${\tilde{g}}_{SR}\left(V\right)={\bar{g}}_{SR}e^{-0.356\left(V+30\right)}/\left(1+e^{-0.356\left(V+30\right)}\right)\text{.}$$

### 2.20 Na dynamics

Intracellular Na dynamics is given by

$$\frac{d{\left[Na^{+}\right]}_{i}}{dt}=\frac{1}{\alpha '}\left(I_{Na}+3I_{NaCa}+3I_{NaK}\right)$$

where the factor 1/α’ converts membrane currents in μA/μF to Na fluxes in units of mM/ms. The conversion factor is given by α’ = 1000α, where α *= Fv_i_/C_m_*.

## 3 Results

### 3.1 EADs directly initiated by *I*_Na_

One possible mechanism of the increased late component of *I*_Na_ is the increased window current mechanism [26, 30, 31]. The peak of the window defined as the cross point of the activation and inactivation curves, which is observed when about 0.16% of Na channels are activated and 0.16% of channels are not inactivated (observed as the value 0.0016 on the ordinal axis in Fig. 1A dashed lines). The conductance at this point is only 0.000256% of the maximum conductance (G_Na_), and thus the maximum steady state *I*_Na_ (conductance × (V_m_ − E_Na_)) is about 0.0057 pA/pF (Fig. 1B dashed line). Note here that the time scale of Na channel inactivation is much shorter (a few ms ∼25 ms) than the time scale of AP (>100 ms). Therefore, the steady state approximation is close to the value of the late component of *I*_Na_ measured during APs.

Next, based on published experimental results for Na channel mutations in the congenital long QT syndrome [26], we shifted the activation curve to lower V_m_ and inactivation curve to higher V_m_ so that the peak of the window becomes between 2 to 3 percent (Fig. 1A solid lines). This window of the Na channel is sufficient to increase the amplitude of *I*_Na_ to ∼1.5 pA/pF which is similar to the other K currents at phases 2 and 3 (Fig. 1B solid line).

**Fig. 2:**
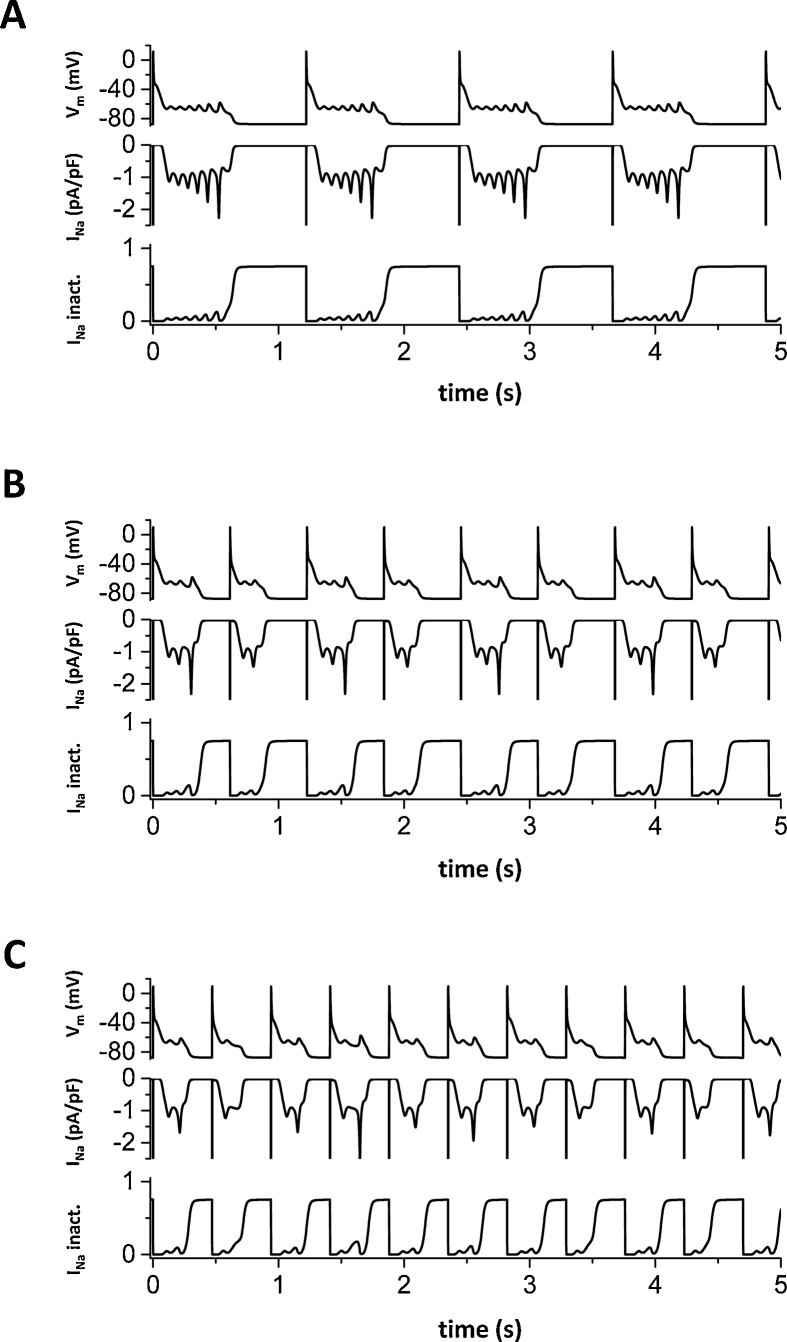
*I*_Na_ mediated EADs*.* Typical EADs due to reactivation of *I*_Na_ in the physiologically detailed model. *I*_CaL_ was blocked (*G*_CaL_ = 0) to show explicitly these EADs are due to reactivation of *I*_Na_. (A) EADs are periodic when pacing cycle length (PCL) = 1220 ms. (B) EADs show period 2 when PCL = 613 ms. (C) EADs are irregular when PCL is 470 ms. Irregular EADs shown here are sensitive to the initial conditions (see Fig. 3).

Using the increased window of *I*_Na_, we show examples of EADs (Fig. 2). Fig. 2A shows periodic EADs. Note that action potentials are slightly different from what we usually observe in experiments because the *I*_CaL_ was completely blocked (*G*_CaL_ = 0). The main purpose of this figure is to show *I*_Na_ by itself can generate EADs. Blocking *I*_CaL_ prevents both EADs due to reactivation of the L-type Ca channel and spontaneous Ca releases from the SR. Therefore, these EADs are solely due to reactivation of the Na channel as it enters the window current range of V_m_. Lower panels in each figure show *I*_Na_ and inactivation (*h* × *j*). These panels show that the Na channel recovers and reactivates along with EADs. These EADs occur around −70 mV, which is lower than the voltage range (−20 ∼ 10 mV) of EADs due to reactivation of the L-type Ca channel (*I*_CaL_-mediated EADs).

**Fig. 3:**
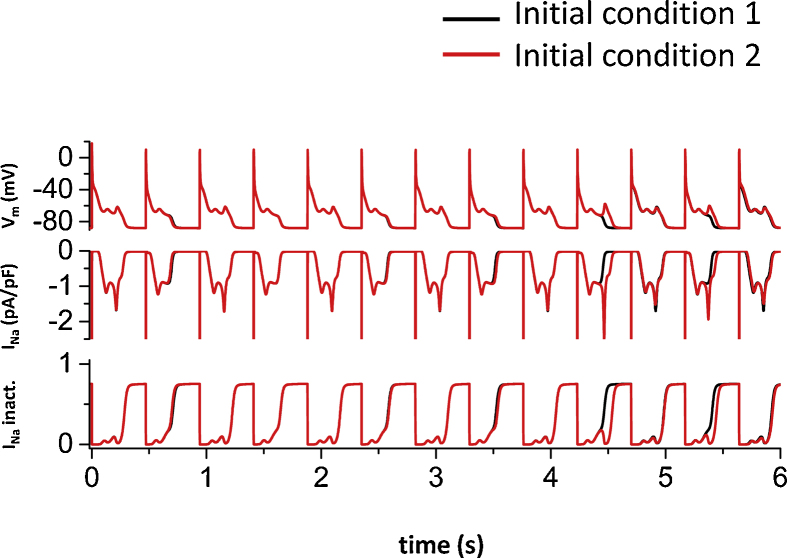
Sensitivity to initial conditions. Irregular EADs with slightly different initial conditions (initial V_m_ in the second simulation (Red line) is 1 mV higher (−86.9 mV) than the first simulation (−87.9 mV) (Black line).

Fig. 2B shows period two EADs, that is AP with three EADs and with two EADs appear alternately. Fig. 2C shows irregular EADs. These irregular EADs are probably chaotic since these EADs are sensitive to the initial conditions, which is a hallmark of chaotic systems. (Fig. 3). When two simulations are performed with slightly different initial conditions, both APs are initially very similar. However, after a couple of beats (about 10 beats in Fig. 3), APs became completely different.

In this study, we used the ventricular cell model. The cell remains excitable around −86 mV and V_m_ stays at the resting potential if there is no external stimulus. This dynamical behavior is clearly different from that of the pacemaker cell, which is oscillatory without stimuli [23, 24].

### 3.2 Mathematical analysis

The condition that the sum of the inward currents is greater than the sum of the outward currents is necessary for depolarization. However, this does not mean that the system always shows oscillatory behavior [32]. In order to elucidate the mechanisms of *I*_Na_ mediated EADs, we reduced the model to 3 variables, which are the membrane potential (*v*), the inactivation gate of the Na channel (*h*), and the total conductivity of K currents (*g_k_*). The set of ordinary differential equations is

$$\{\begin{array}{c} \frac{dv}{dt}=-\left(g_{Na}m_{\infty }^{3}h^{2}\left(v-e_{na}\right)+g_{k}\left(v-e_{k}\right)\right),\\ \begin{array}{c} \frac{dh}{dt}=\frac{h_{\infty }-h}{\tau_{h}},\\ \frac{dg_{k}}{dt}=\frac{gk_{\infty }-gk}{\tau_{k}}, \end{array} \end{array}$$

$$gk_{\infty }=1-\exp\left(-3\frac{v+100}{200}\right)\text{,}$$

$\tau_{k}=300\;\text{ms}$.

The first equation shows V_m_ change due to the simplified currents of *I*_Na_ and *I*_K_. Inactivation gates *h* and *j* are almost identical except for their time constants. Here we reduce them as simply *h*^2^. The smaller *τ*_*h*_ gives faster oscillations. However, the fixed points remain the same. The third equation represents the fact that K currents (*I*_Ks_, *I*_Kr_ etc) increase with time and bring V_m_ back to the resting V_m_. This generic K current was adopted from the simplified model of the cardiac action potential by Echebarria and Karma [33].

**Fig. 4:**
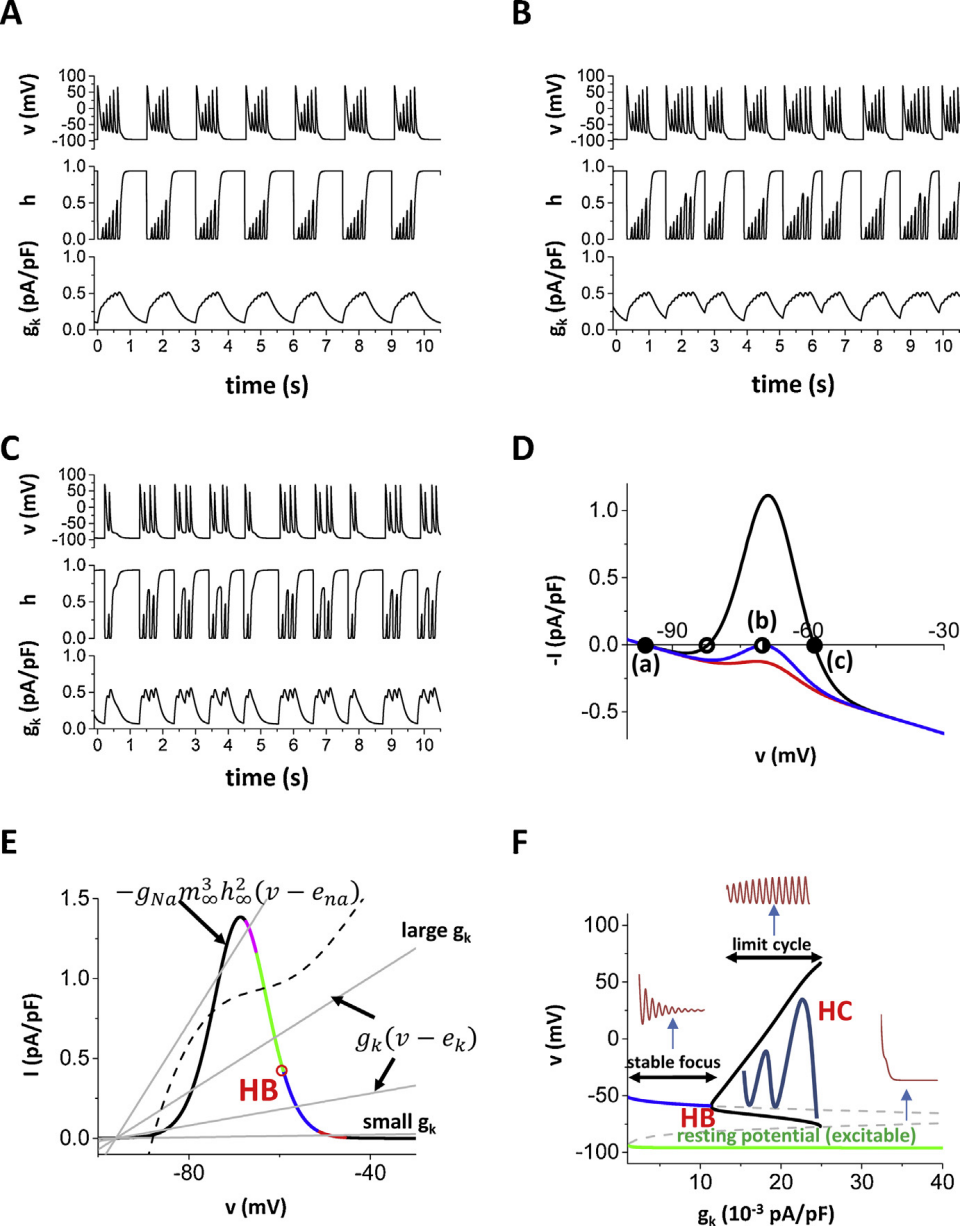
Eigenvalues and dynamical behaviors. (A) EADs in the simplified 3-variable model. PCL is 1500 ms. (B) Period 3 EADs. (C) Irregular (probably chaotic) EADs. (D) Fixed points. With the normal *I*_Na_, the system has only one fixed point (red). As *I*_Na_ becomes larger (red → blue → black), three fixed points appear. (E) Eigenvalues change as *I*_K_ is increased. Red: negative real (e.g. −0.0242 at *g_k_* = 0.001), Blue: negative complex (e.g. −0.005545 ± 0.081 at *g_k_* = 0.01), Green: positive complex (e.g 0.0293 ± 0.09 at *g_k_* = 0.02), Magenta: positive real (e.g. 0.1405 at *g_k_* = 0.04). HB: Hopf bifurcation. Dashed line shows sum of K currents of the physiologically detailed model. (F) Bifurcation diagram. Green line: resting state (stable). The system is always excitable from here. Blue line: stable state (stable focus). Black brunches: maximum and minimum *v* of the limit cycle. Dashed line: unstable steady state. HB: Hopf bifurcation. HC: homoclinic bifurcation.

This reduced model shows both excitability (i.e. action potential) and oscillatory (i.e. EADs) (Fig. 4A). EADs can be periodic (Fig. 4A), period 2, period 3 (Fig. 4B) and even chaotic (Fig. 4C). Steady state (−*I*) vs. V curves are shown in Fig. 4D. Here we chose (*−I*) instead of *I* according to standard nonlinear dynamics notation (in contrast to standard electrophysiology nomenclature). If the inward window current is small (red curve), there is only one fixed point (a, filled circle), which is the resting potential of the ventricular cell. This system shows only excitability at the resting potential. As the window current is increased, another fixed point (b, half-filled circle) appears (blue curve) and then, at higher window current, a third fixed point (c, filled circle) appears.

In order to understand the oscillation around the upper fixed point, we consider the two variable system of *v* and *h*. Since *g_k_* is the slowest variable of this system, we can identify the behavior of the *v*-*h* system for each *g*_*k*_ value using eigenvalues of the *v*-*h* system described by the following matrix.

$$\left(\begin{array}{cc} \frac{\partial F}{\partial v} & \frac{\partial F}{\partial h}\\ \frac{\partial G}{\partial v} & \frac{\partial G}{\partial h} \end{array}\right)\text{,}$$

where

$$\{\begin{array}{c} F=-\left(g_{Na}m_{\infty }^{3}h^{2}\left(v-e_{na}\right)+g_{k}\left(v-e_{k}\right)\right),\\ G=\frac{h_{\infty }-h}{\tau_{h}}, \end{array}$$

If the eigenvalues of the system are complex, the system of *v* and *h* is oscillatory. In other words, having complex eigenvalues is the necessary condition for EADs. In the simplified model, *I*_K_ is shown as a straight line (no rectification) in Fig. 4E. At the cross point of this line and −*I*_Na_ is the stable fixed point (dv/dt = 0, the system does not move from this point). In other words, the outward current is equal to the inward current (|*I*_Na_| = |*I*_K_|). We compute eigenvalues, which determine the stability of the system, for the fixed point. Depending on the value of g_k_, eigenvalues can be real positive, real negative, complex positive, or complex negative. Corresponding biological phenomena are

1) real negative → prolongation of AP without oscillation
2) complex negative → decaying EADs
3) complex positive → growing EADs
4) real positive → repolarization to the resting potential (no EAD)

In Fig. 4E eigenvalues are shown in different colors. If *I*_K_, which is the conductance (*g_k_*) times the driving force (*v* − *e_k_*), is very small, the larger eigenvalue is real negative. In this case, the fixed point is an attractor without oscillations. Since this requires very small *I*_K_, this may not occur physiologically. If *I*_K_ is slightly larger (red part), then the eigenvalues are complex negative and the fixed point is an attractor with V_m_ oscillations (damped EADs) (stable focus). When *g_k_* is included as a third variable, *I*_K_ increases as time goes. The eigenvalues become complex positive (green part) from complex negative. At this point (red circle in Fig. 4E and ‘HB’ in Fig. 4F), Hopf bifurcation occurs and the EAD amplitude grows. As *I*_K_ becomes sufficiently large, homoclinic bifurcation occurs (‘HC’ in Fig. 4F) and V_m_ goes back to the resting potential.

**Fig. 5:**
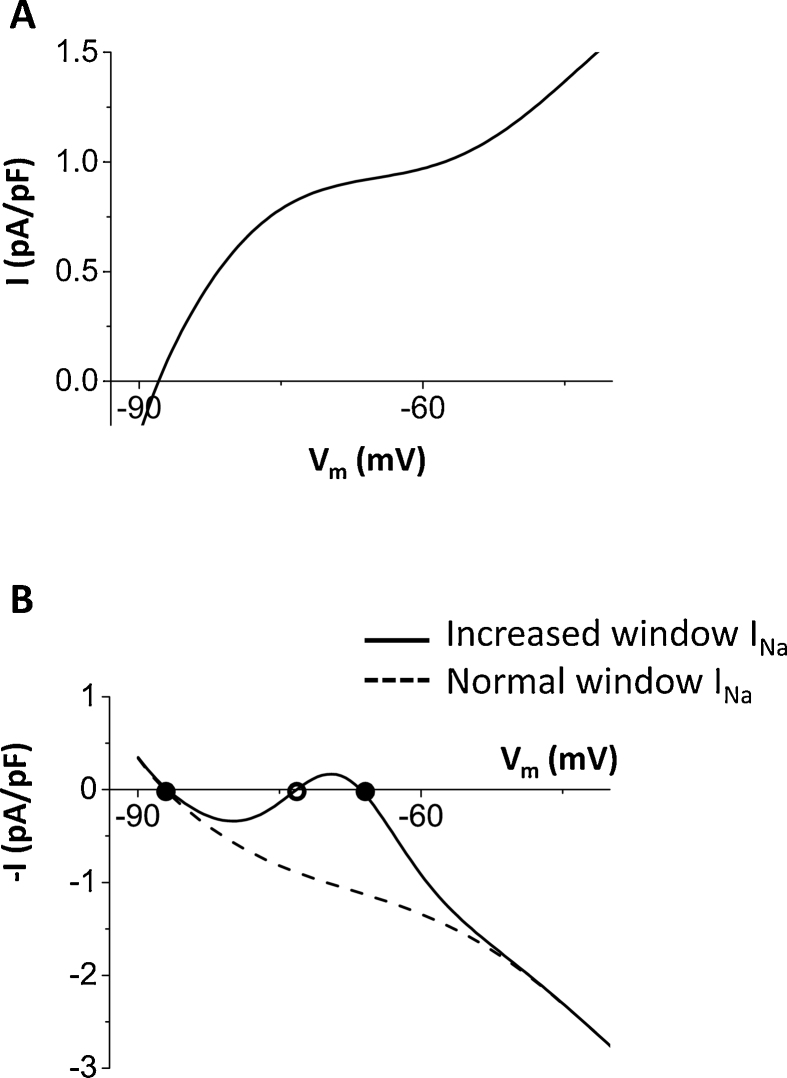
Fixed points of the physiologically detailed model. (A) The generic K current in the simplified model represents the total K current in the physiological model. Total K current vs voltage. Total K current = *I*_Ks_ + *I*_Kr_ + *I*_K1_ + *I*_to_ + *I*_NaK_. (B) Total current vs voltage. Solid line: total current with the increased window *I*_Na_. Dashed line: total current with the normal window *I*_Na_.

The I–V curve of the simplified K current is slightly different from the I–V curve of the total K current in the physiological model (Fig. 5). This simplification may affect the results quantitatively but will not qualitatively as far as the *I*_Na_ exceeds the sum of K currents and Hopf bifurcation occurs. Fig. 5A shows the total K current in the physiologically detailed model. With the normal window current, there is only one fixed point (Fig. 5B dashed line). However, when the window current is increased, three fixed points appear (Fig. 5B solid line). EADs in the physiologically detailed model are oscillation around the rightmost fixed point.

**Fig. 6:**
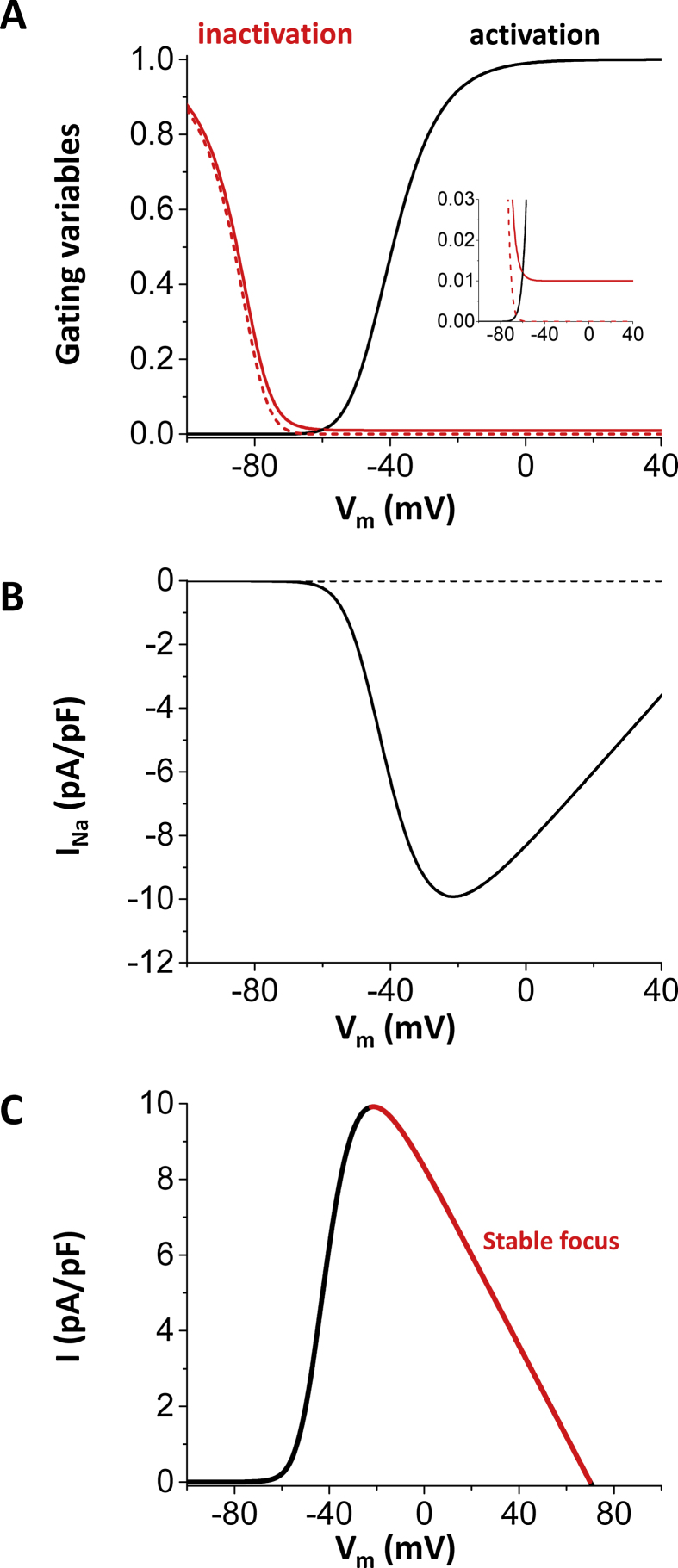
Non-inactivating *I*_Na_ does not cause EADs. (A) Activation and inactivation curves. Solid lines: non-inactivating Na channel. Dashed lines: normal Na channel. (B) Steady state current. Solid lines: non-inactivating Na current. Dashed lines: normal Na current. (C) Eigenvalues when the mechanism of the late component of *I*_Na_ is non-inactivation of the Na channel.

Non-inactivation also increases the late component of *I*_Na_. However, when the late component of *I*_Na_ is due to non-inactivating current, although three fixed points can appear, the eigenvalues are always real negative, which indicates no oscillation of V_m_ (Fig. 6). For example, when 1% of channels are non-inactivating (Fig. 6A), steady state *I*_Na_ becomes extremely large (Fig. 6B). However, this will not cause EADs since eigenvalues are always real negative (stable focus) although it prolongs the action potential (Fig. 6C).

Therefore, EADs due to reactivation of *I*_Na_ will not occur in this case although this late component of *I*_Na_ may set up the conditions for *I*_CaL_-mediated EADs by reducing repolarization reserve and EADs due to spontaneous Ca releases by increasing [Na]_i_, which leads to Ca overload.

### 3.3 Interplay of *I*_Na_ and *I*_CaL_ mediated EADs

**Fig. 7:**
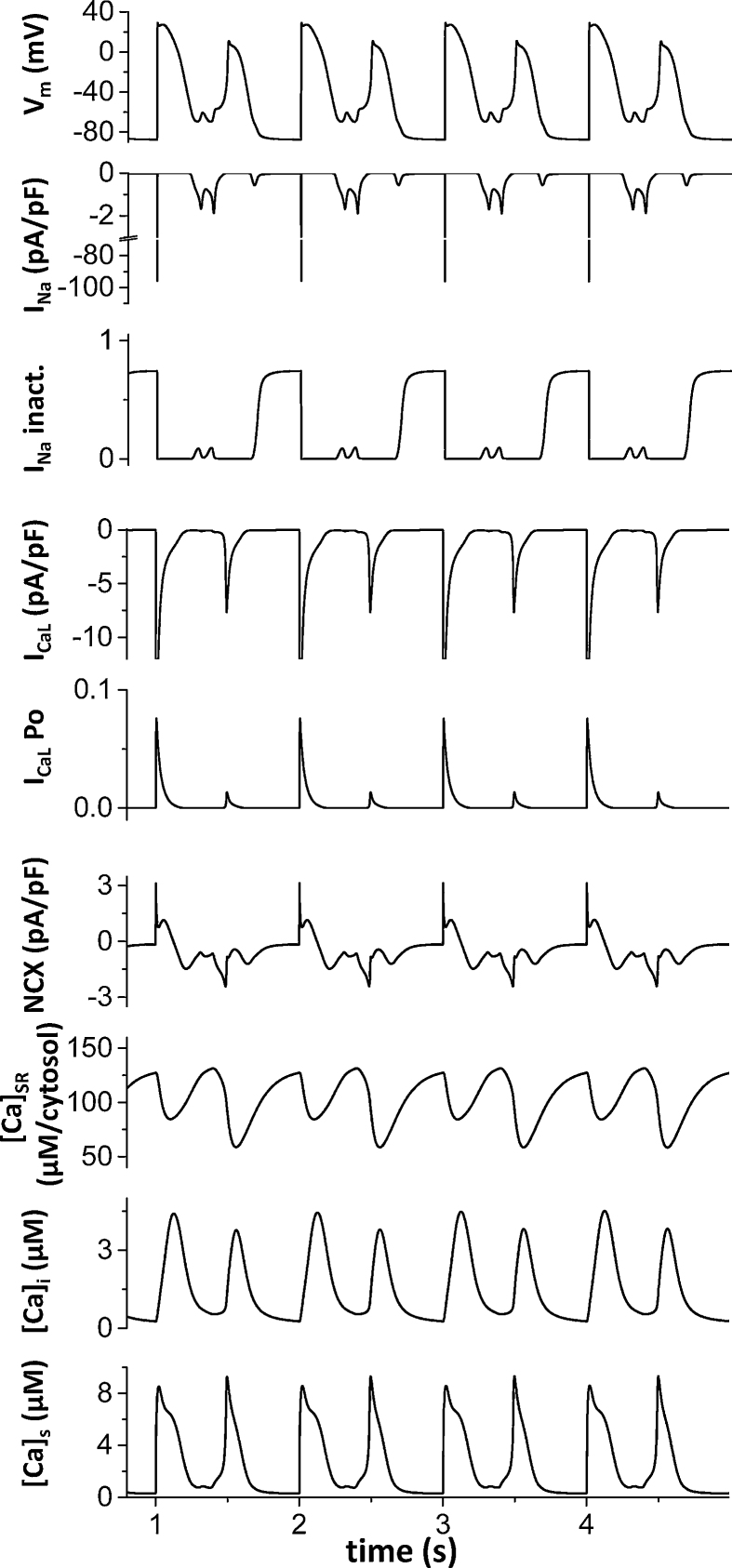
Interplay of *I*_Na_ and *I*_CaL_ mediated EADs. Reactivation of the Na channel promotes reactivation of the L-type Ca channel. Example of EADs when both *I*_CaL_ and *I*_Na_ present. The membrane potential, Na current, Na inactivation, L-type Ca current, L-type Ca channel open probability, NCX, SR [Ca], cytosolic [Ca], and submembrane [Ca] are shown. The *I*_Na_ mediated EADs occurred prior to the *I*_CaL_ mediated EADs. Ca entry via *I*_CaL_ also activates NCX and further promotes EADs. Here μM/cytosol means μM *v_sr_/v_i_*, where *v_sr_* is the SR volume and *v*i is the cell volume.

In Fig. 2, in order to explore the possibility of *I*_Na_ mediated EADs, *I*_CaL_ was blocked. Fig. 7 shows how *I*_Na_ mediated EADs directly promote *I*_CaL_ mediated EADs in the presence of *I*_Na_ and *I*_CaL_. Although we cannot say which the cause of EADs is since these are nonlinearly coupled in the system, Fig. 7 clearly shows that reopening of the Na channel precedes reopening of the L-type Ca channel. In this simulation, we used the normal (healthy) L-type Ca channel [25], which is much more difficult to generate EADs than the L-type Ca channel under administration of ISO[32] or H_2_O_2_ [6]. When V_m_ became around −70 mV, the Na channel was reactivated first. This caused elevation of V_m_, which promoted reactivation of the healthy L-type Ca channel. Ca entry through the L-type Ca channels also helps depolarization via NCX and further promotes EADs.

## 4 Discussion

In this study, we showed that *I*_Na_ by itself is able to generate EADs. The dynamical mechanism is oscillation in the *I*_Na_-*I*_K_ system around the higher V_m_ fixed point, which is distinguished from the oscillation in the pacemaker cell (oscillation around the single fixed point). *I*_Na_, especially the late component of *I*_Na_ has been recognized as an important player to set up the conditions for EADs by reducing repolarization reserve and increasing intracellular Na concentration, which leads to Ca overload. However, *I*_Na_ itself has not been considered as a direct initiator of EADs. Under normal conditions, the late component of *I*_Na_ is so small (Fig. 1B dashed line) that the amplitude of *I*_Na_ cannot be larger than the sum of K currents at phases 2 and 3, and therefore, *I*_Na_ itself cannot initiate EADs. However, under pathological conditions such as heart failure [34, 35, 36] and myocardial ischemia [37, 38], large late *I*_Na_ has been observed. Recent experimental study by Horvath et al. showed that *I*_Na_ is as large (∼1 pA/pF) as the other Ca and K currents. We reconstructed *I*_Na_ based on activation and inactivation curves (Fig. 1A) measured by Wang et al. and the amplitude of *I*_Na_ predicted by the model gives similar amplitude (Fig. 1B solid line). This implies that *I*_Na_ may overcome the sum of *I*_K_ and depolarize V_m_ during AP without the help of the other inward currents such as *I*_CaL_, NCX, non-specific Ca-activated cation current, especially, when *I*_K_ become small under pathological conditions and/or administration of K channel blockers. Using the physiologically detailed model of the ventricular action potential, we showed *I*_Na_ mediated EADs (Fig. 2). As we have shown in *I*_CaL_-mediated EADs [6], *I*_Na_-mediated EADs can be periodic (Fig. 2A), period-2 (Fig. 2B), and even chaotic (Fig. 2C).

We have shown the mechanisms of *I*_CaL_-mediated EADs [32, 39]. Also, bursting behaviors are widely observed and studied in many biological systems [20, 21, 22]. In this study, we reduced the physiologically detailed model to the 3-variable model and analyzed the dynamical mechanism of *I*_Na_ mediated EADs. This methodology has been used in theoretical neuroscience to understand underlying mechanisms [22, 40]. We have also used this type of analysis for *I*_CaL_-mediated EADs [32, 39]. For the formation of EADs, positive feedback processes such as *I*_CaL_ or Ca induced Ca release from the SR are necessary. In addition to them, *I*_Na_ has a positive feedback process since more Na channels open as V_m_ elevates. In this study, we showed *I*_Na_-mediated EADs due to the Na channel reactivation. The EAD, namely the oscillation of V_m_ at phases 2 and/or 3, in this case is distinguished from the V_m_ oscillation in the pacemaker cell [23, 24]. In the model of the voltage clock of the pacemaker cell, the system has only one fixed point, which is unstable, gives V_m_ oscillation. On the other hand, the model shown in this study has three fixed points as the window current is increased. *I*_Na_ mediated EADs are due to oscillation around the higher V_m_ fixed point. The lower fixed point (the resting potential) is still stable and the system shows excitability.

The Na channel can reactivate without large window current if there is another positive feedback mechanism, which helps reactivation of the Na channel. For example, Edward et al. have shown that reactivation of Na channel when SR Ca release occurs [41, 42]. In these cases, the higher V_m_ fixed point is not necessary. Stimulation such as spontaneous SR Ca release via NCX activates the Na channel from the lower V_m_ fixed point.

The sustained Na current can also be due to non-inactivation of the Na channel. However, our analysis shows that AP will be simply prolonged and EADs will not occur (Fig. 6) in this case. The prolongation of AP without EADs is also observed with late component of the Na current experimentally [19]. In this case, although the Na channel will not reactivate, prolongation can promote I_CaL_-mediated EADs and Ca overload.

In order to increase the window current, activation and inactivation curves are shifted. The amplitude of the upstroke of AP becomes larger since activation occurs at the lower potential and inactivation requires higher potential. Zhao et al. have shown that smaller amplitude of the upstroke promotes EADs [43]. In our case, the amplitude is smaller with the normal *I*_Na_. However, there is only one fixed point with the normal *I*_Na_ (Fig. 4D red curve, Fig. 5 dashed curve). Therefore, even if the amplitude of the upstroke is smaller, EADs will not occur with the normal *I*_Na_ unless K currents are unphysiologically small.

In the case of *I*_CaL_-mediated EADs, *I*_CaL_ is responsible only for oscillation at phases 2 and/or 3. On the other hand, in the case of *I*_Na_-mediated EADs, *I*_Na_ is responsible for both excitation and oscillation. In addition, *I*_CaL_-mediated EADs occur around −20 ∼ 10 mV. In some experiments, EADs are observed at more negative V_m_ than the reactivation V_m_ of the L-type Ca channel [42, 44]. Damiano and Rosen observed more EADs as the PCL becomes longer [44]. As the PCL becomes longer, SR Ca load becomes smaller and EADs due to spontaneous Ca releases occur less in large mammalian ventricular cells. Therefore, if the mechanism of EADs is due to spontaneous Ca releases, EADs should occur less as the PCL becomes longer. In addition, if the mechanism of EADs is due to reactivation of the L-type Ca channel, EADs should occur at more positive voltage. This experimental observation suggests that these EADs are due to Na channel reactivation and consistent with our results (Fig. 2, more EADs with longer PCLs). Our mechanism may explain these EADs although we need additional experiments to differentiate them more explicitly from EADs caused by the other mechanisms.

Recently, the current through the Nav1.8 channel has been considered to be a possible mechanism of *I*_NaL_ in cardiac cells [45, 46]. This channel also has a positive feedback process like Nav1.5 and would similarly cause *I*_Na_ mediated EADs. However, EADs would occur a much higher V_m_ range, because for Nav1.8 the window current V_m_ range is closer to that of the L-type Ca channel [45].

## Declarations

### Author contribution statement

Daisuke Sato: Conceived and designed the experiments; Performed the experiments; Analyzed and interpreted the data; Contributed reagents, materials, analysis tools or data; Wrote the paper.

Colleen E. Clancy, Donald M. Bers: Conceived and designed the experiments; Analyzed and interpreted the data; Contributed reagents, materials, analysis tools or data; Wrote the paper.

### Competing interest statement

The authors declare no conflict of interest.

### Funding statement

This work was supported by National Institutes of Health grant K99/R00-HL111334, American Heart Association Grant-in-Aid 16GRNT31300018, and Amazon AWS Cloud Credits for Research (D.S.), National Institutes of Health grants U01-HL126273 and R01-HL128170 (C.E.C.), and National Institutes of Health grants R37-HL30077 and R01-HL105242 (D.M.B.)

### Additional Information

No additional information is available for this paper.
